# The risk of contralateral breast cancer in patients from *BRCA1/2 *negative high risk families as compared to patients from *BRCA1 *or *BRCA2 *positive families: a retrospective cohort study

**DOI:** 10.1186/bcr3369

**Published:** 2012-12-07

**Authors:** Kerstin Rhiem, Christoph Engel, Monika Graeser, Silke Zachariae, Karin Kast, Marion Kiechle, Nina Ditsch, Wolfgang Janni, Christoph Mundhenke, Michael Golatta, Dominic Varga, Sabine Preisler-Adams, Tilman Heinrich, Ulrich Bick, Dorothea Gadzicki, Susanne Briest, Alfons Meindl, Rita K Schmutzler

**Affiliations:** 1 University Hospital Cologne, Center of Familial Breast and Ovarian Cancer, Kerpener Str. 34, Cologne 50931, Germany; 2 University of Leipzig, Institute for Medical Informatics, Statistics and Epidemiology, Haertelstr. 16-18, Leipzig 04107, Germany; 3 University Hospital Dresden, Department of Gynecology and Obstetrics, Fetscherstr. 74, Dresden 01307, Germany; 4 Technical University Munich, Department of Gynecology and Obstetrics, Division of Tumor Genetics, Ismaningerstr. 22, Munich 81675, Germany; 5 University Hospital Grosshadern, Department of Gynecology and Obstetrics, Marchioninistr. 15, Munich 81377, Germany; 6 University Hospital Duesseldorf, Department of Gynecology and Obstetrics, Moorenstr. 5, Duesseldorf 40225, Germany; 7 University Hospital Kiel, Department of Gynecology and Obstetrics, Arnold-Heller-Str. 3, Kiel 24105 Kiel, Germany; 8 University Hospital Heidelberg, Department of Gynecology and Obstetrics, Voßstr. 9, Heidelberg 69115, Germany; 9 University Hospital Ulm, Department of Gynecology and Obstetrics, Prittwitzst. 43, Ulm 89075, Germany; 10 University Hospital Muenster, Department of Gynecology and Obstetrics, Albert-Schweitzer-Campus 1, 48149 Muenster, Germany; 11 University of Wuerzburg, Department of Human Genetics, Biocenter Am Hubland, Wuerzburg 97074, Germany; 12 University Hospital Berlin, Charité Campus Mitte, Department of Radiology, Charitéplatz 1, Berlin 10117 Berlin, Germany; 13 Hannover Medical School, Institute of Cell and Molecular Pathology, Carl-Neuberg-Str. 1, Hannover 30625, Germany; 14 University Hospital Leipzig, Department of Gynecology and Obstetrics, Liebigstr. 20a, Leipzig 04103, Germany

## Abstract

**Introduction:**

While it has been reported that the risk of contralateral breast cancer in patients from *BRCA1 *or *BRCA2 *positive families is elevated, little is known about contralateral breast cancer risk in patients from high risk families that tested negative for *BRCA1/2 *mutations.

**Methods:**

A retrospective, multicenter cohort study was performed from 1996 to 2011 and comprised 6,235 women with unilateral breast cancer from 6,230 high risk families that had tested positive for *BRCA1 *(*n *= 1,154) or *BRCA2 *(*n *= 575) mutations or tested negative (*n *= 4,501). Cumulative contralateral breast cancer risks were calculated using the Kaplan-Meier product-limit method and were compared between groups using the log-rank test. Cox regression analysis was applied to assess the impact of the age at first breast cancer and the familial history stratified by mutation status.

**Results:**

The cumulative risk of contralateral breast cancer 25 years after first breast cancer was 44.1% (95%CI, 37.6% to 50.6%) for patients from *BRCA1 *positive families, 33.5% (95%CI, 22.4% to 44.7%) for patients from *BRCA2 *positive families and 17.2% (95%CI, 14.5% to 19.9%) for patients from families that tested negative for *BRCA1/2 *mutations. Younger age at first breast cancer was associated with a higher risk of contralateral breast cancer. For women who had their first breast cancer before the age of 40 years, the cumulative risk of contralateral breast cancer after 25 years was 55.1% for *BRCA1*, 38.4% for *BRCA2*, and 28.4% for patients from *BRCA1/2 *negative families. If the first breast cancer was diagnosed at the age of 50 or later, 25-year cumulative risks were 21.6% for *BRCA1*, 15.5% for *BRCA2*, and 12.9% for *BRCA1/2 *negative families.

**Conclusions:**

Contralateral breast cancer risk in patients from high risk families that tested negative for *BRCA1/2 *mutations is similar to the risk in patients with sporadic breast cancer. Thus, the mutation status should guide decision making for contralateral mastectomy.

## Introduction

Women carrying a deleterious *BRCA1 *or *BRCA2 *mutation not only face a strongly elevated lifetime risk for the development of breast and ovarian cancer but also for a second breast cancer [1,2]. The majority of second primaries develop in the contralateral breast while ipsilateral breast cancer is not significantly increased [3,4]. Estimates for contralateral breast cancer range from 15% to 40% within 10 years [1,3,5-10]. Due to this wide range of risk estimates, it is clinically important to identify predictive factors. In a retrospective cohort study comprising 2,020 women with unilateral breast cancer from 978 families with a *BRCA1 *or *BRCA2 *mutation, we could show that contralateral breast cancer depends on the affected *BRCA *gene and age at onset of the first breast cancer [5]. In a recent retrospective update on 810 breast cancer patients from *BRCA1/2 *positive families, Metcalfe *et al. *as well as previous studies also showed that prophylactic bilateral salpingo-oophorectomy (PBSO) under the age of 50 years reduces the risk of contralateral breast cancer by half [9,10]. They further demonstrated that as the number of first degree relatives under the age of 50 years with breast cancer increases the risk of contralateral breast cancer also increases. Data on other factors that may modify contralateral breast cancer risk, for example tamoxifen use, are inconsistent [10,11].

While the risk for second primaries has been studied in *BRCA1 *or *BRCA2 *mutation carriers, preliminary data indicate that the risk of contralateral breast cancer is not significantly elevated in patients with familial breast cancer, who tested negative for *BRCA1/2 *mutations [12,13]. Despite these data and although the latter group accounts for the majority of women with familial breast cancer, there is a rising demand for prophylactic bilateral or prophylactic contralateral mastectomy in these women [14,15]. To question the need for this prophylactic approach, in this article we extended our previously published data on contralateral breast cancer risks and predictive factors in two dimensions. First, we updated the number of analyzed women from families with *BRCA1 *or *BRCA2 *mutations. Second, we included women from non-*BRCA1/2 *high risk families in the analysis for the first time. These risk estimates can be used for counselling in order to allow women to make a non-directive and informed decision on the extent of surgical treatment and secondary prevention.

## Methods

### German Consortium for Hereditary Breast and Ovarian Cancer

Data were collected within the German Consortium for Hereditary Breast and Ovarian Cancer which comprises 12 university based centers, as previously described [5]. Inclusion criteria and methods for genetic testing are described elsewhere [16,17]. It is worth mentioning that 24% of all families that fulfill the inclusion criteria of the German Consortium for Hereditary Breast and Ovarian Cancer tested positive for a deleterious *BRCA1 *or *BRCA2 *mutation which reflects stringent inclusion criteria [16,17]. The study has been approved by the institutional review boards of all participating centers. All patients gave their written informed consent prior to study inclusion.

From 1996 to July 2011 a total of 8,733 families were registered and tested positive for *BRCA1 *(*n *= 1,743) or *BRCA2 *(*n *= 818) mutations or tested negative (*n *= 6,172). For this updated retrospective cohort study all index cases and their first- and second-degree relatives with a history of unilateral breast cancer diagnosed after 1960 were selected (12,897 patients from 6,364 families). Individuals were excluded if their first breast cancer diagnosis was bilateral (synchronous bilateral breast cancer). Individuals were only selected from that branch of the family in which the pathogenic mutation was detected or familial clustering of cancers was observed. All index patients, defined as the earliest accessible affected family member and diagnosed with breast or ovarian cancer, were tested for *BRCA1/2 *mutations. Of the selected relatives of *BRCA1/2 *mutation carriers, 328 (16%) could be proven to be carriers. Forty relatives who tested negative for the known mutation in the family were considered phenocopies and were excluded. All other relatives, whose mutation status could not be determined, were considered putative carriers. An additional 392 patients (3.0%) were excluded due to insufficient information regarding age at cancer events or surgery (for example, bilateral mastectomy). In summary, 12,465 women with unilateral first breast cancer diagnosis were included in the present analysis, comprising 6,230 index patients and 6,235 relatives. Medical and pathological records could be obtained from 83% of the index patients and 47% of the relatives. For all other individuals information about medical history was obtained through structured interviews. The study was performed retrospectively. Prospective follow-up after recruitment is not considered in this analysis. For the analysis, patients were followed from their first unilateral breast cancer until contralateral breast cancer or censoring.

For the comparison of contralateral breast cancer risk in *BRCA1 *and *2 *mutation carriers and *BRCA1/2*-negative women at high risk we used data from breast cancer registries [18,19].

### Statistical analysis

*BRCA1*, *BRCA2 *and non-*BRCA1/2 *families were analyzed as previously described [5]. In summary, cumulative contralateral breast cancer risks were calculated using the Kaplan-Meier product-limit method and compared between groups using the log-rank test. Cox proportional hazards regression was used to analyze the association with potential risk factors by estimation of hazard ratios (HR) and their 95% CIs. All subjects were censored at the time of second unilateral breast cancer, ovarian cancer, bilateral mastectomy, death, or last observation, whichever occurred first. We censored at unilateral breast cancer and ovarian cancer because an effect of chemotherapy for these cancers on the risk of subsequent contralateral breast cancer can not be excluded. All reported *P*-values are two-sided. *P*-values below 0.05 were considered statistically significant. IBM SPSS Statistics 20.0.0.1 was used for all data analyses.

## Results

### Characteristics of the study population

**Figure 1:**
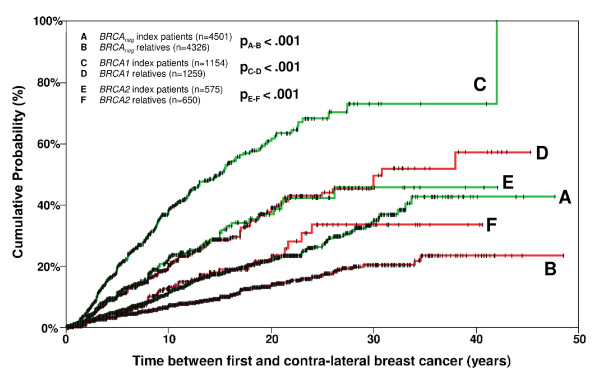
**Cumulative risk of contra-lateral breast cancer after first breast cancer in index cases versus relatives from *BRCA1/2 *positive families and *BRCA1/2 *negative families**.

Basic characteristics of the index patients and their relatives are shown in Table 1. Index patients had a younger median age at first unilateral breast cancer and a considerably higher risk for contralateral breast cancer than their relatives (Figure 1). This was true for the whole study cohort as well as for the three subgroups, that is, *BRCA1 *positive, *BRCA2 *positive and *BRCA1/2 *negative families, and is most likely due to the selection of index patients based on clinical criteria, that is, DNA testing was preferentially performed in those patients with clinical phenotypes that were more indicative of a *BRCA *mutation. Thus, in order to avoid overestimating the risks of contralateral breast cancer, index patients were excluded from further analyses.

**Table 1:** Basic characteristics of relatives and index patients from *BRCA *positive and negative families.

	Relatives of index patients	Index patients
Number of patients	6,235	6,230
Mutation status, number of patients		
*BRCA1 *pathogenic mutation	213	1,154
*BRCA2 *pathogenic mutation	106	575
BRCA negative	4,326	4,501
not tested, *BRCA1 *family	1,046	-
not tested, *BRCA2 *family	544	-
Patients with contralateral breast cancer		
patients from *BRCA1 *families	193	304
patients from *BRCA2 *families	56	84
patients from BRCA negative families	253	349
Median year of birth (IQR)		
patients from *BRCA1 *families	1,943 (1933-1955)	1,960 (1952-1968)
patients from *BRCA2 *families	1,939 (1928-1952)	1,957 (1945-1965)
patients from BRCA negative families	1,936 (1926-1946)	1,955 (1944-1964)
Median age at first breast cancer (IQR)		
patients from *BRCA1 *families	43.5 (37.5-51.5)	38.2 (32.8-44.4)
patients from *BRCA2 *families	48.1 (40.4-58.5)	42.5 (36.4-49.4)
patients from BRCA negative families	53.6 (45.3-63.9)	44.9 (37.9-51.4)
Median age at contralateral breast cancer (IQR)		
patients from *BRCA1 *families	47.7 (40.1-55.5)	43.5 (37.7-50.5)
patients from *BRCA2 *families	53.1 (44.7-62.6)	47.9 (42.7-55.7)
patients from BRCA negative families	56.0 (48.5-66.6)	51.6 (45.3-59.0)

IQR, interquartile range.

### Risk of contralateral breast cancer depending on mutation status

**Figure 2:**
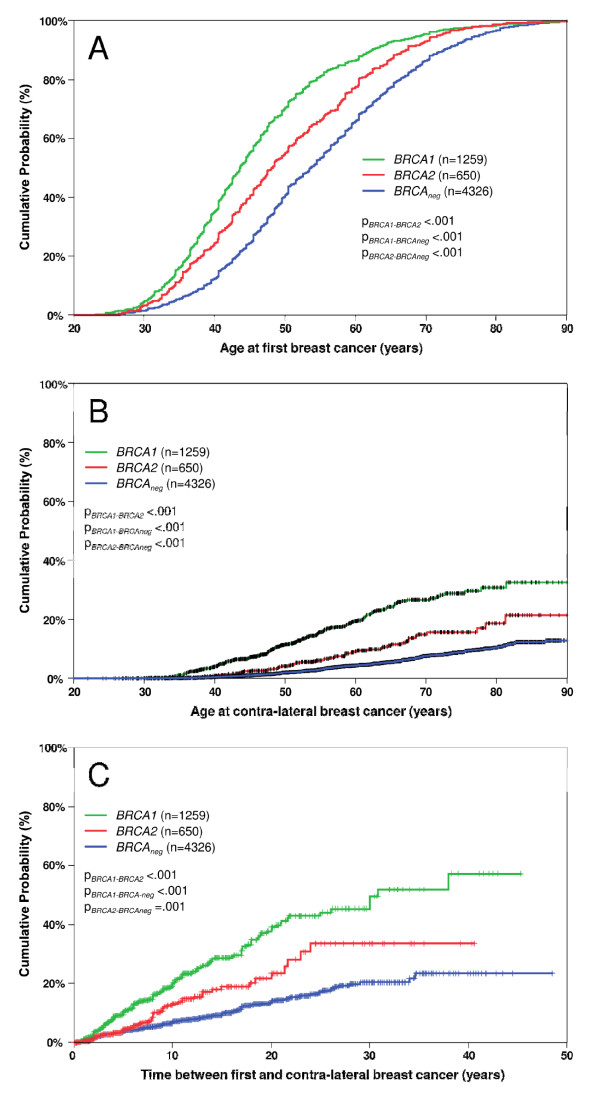
**Effect of mutation status on cancer risk**. Panel **A **shows the cumulative distribution of the age of diagnosis at first breast cancer. Panel **B **depicts the age of diagnosis at contralateral breast cancer. Panel **C **represents the cumulative risk of contralateral breast cancer after first breast cancer. Index patients were excluded.

In 6,235 relatives of index patients, 502 contralateral breast cancers were observed. The total observation time from first breast cancer until contralateral breast cancer or censoring was 48,390 person-years. Figure 2A shows the distribution of age at first breast cancer for relatives of families with *BRCA1 *and *BRCA2 *mutations and of *BRCA1/2 *negative families. Relatives of families with *BRCA1 *mutations were significantly younger at first breast cancers than those from families with *BRCA2 *mutations (*P *< 0.001) and both were significantly younger than patients from *BRCA1/2 *negative families (*P *< 0.001 and *P *< 0.001, respectively). Likewise, the age at contralateral breast cancer was also significantly lower in the *BRCA1 *group than the *BRCA2 *group (*P *< 0.001) and the *BRCA1/2 *negative group (*P *< 0.001) (Figure 2B). Analyses of the time from first breast cancer to contralateral breast cancer showed that contralateral breast cancer risk was significantly higher in women from families with a *BRCA1 *mutation compared to women from families with a *BRCA2 *mutation and compared to women from families without a *BRCA *mutation (Figure 2C). Members of families with *BRCA1 *mutations had a threefold (95%CI, 2.5 to 3.6) higher risk of contralateral breast cancer than members of families without *BRCA1/2 *mutations. For members of families with *BRCA2 *mutations the risk was 1.6-fold (95% CI, 1.2 to 2.2) higher than the risk for members of families without *BRCA1/2 *mutations. The 25-year cumulative risk of contralateral breast cancer after first breast cancer was 44.1% (95%CI, 37.6% to 50.6%) for relatives from families with a *BRCA1 *mutation, 33.5% (95%CI, 22.4% to 44.7%) for relatives from families with a *BRCA2 *mutation and 17.2% (95%CI, 14.5% to 19.9%) for relatives from families without a *BRCA1/2 *mutation.

### Risk of contralateral breast cancer depending on age at first breast cancer

**Figure 3:**
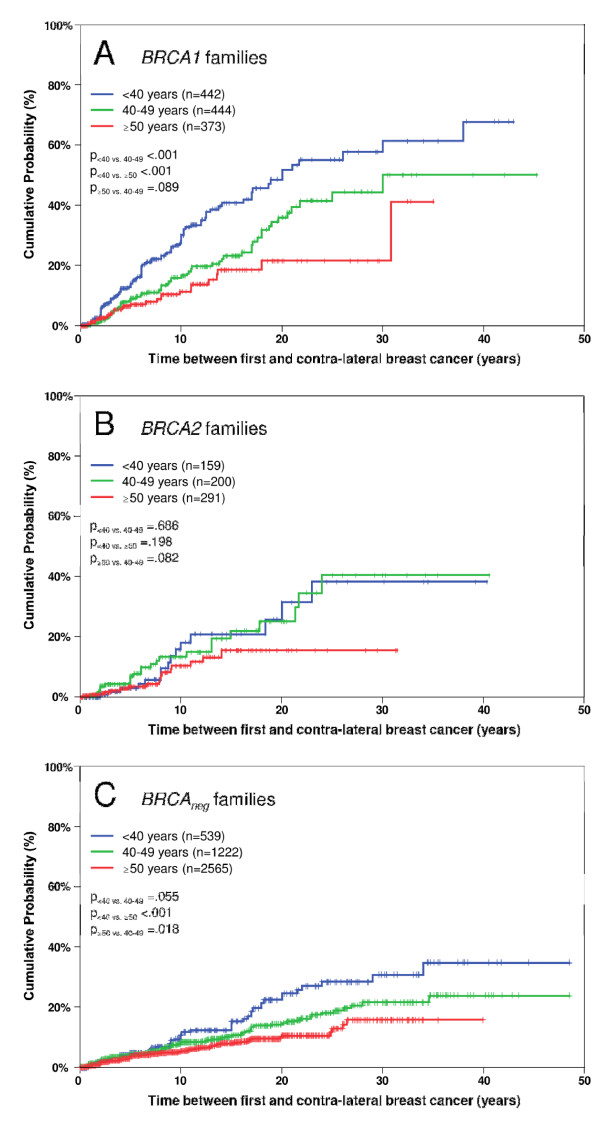
**Effect of age at first breast cancer on contralateral breast cancer risk**. All panels show the cumulative risk of contralateral breast cancer after first breast cancer. Panel **A**: relatives from *BRCA1 *positive families, Panel **B**: relatives from *BRCA2 *positive families, Panel **C**: relatives from *BRCA1/2 *negative families. Index patients were excluded.

Younger age at first breast cancer was associated with a significantly higher risk of contralateral breast cancer in patients from *BRCA1 *positive and *BRCA1/2 *negative families and a trend was observed in patients from *BRCA2 *positive families (Figures 3A, B and C). For instance, after 25 years 55.1% (95%CI, 45.4% to 64.9%) of patients from *BRCA1 *positive families, 38.4% (95% CI, 18.5% to 58.2%) of patients from *BRCA2 *families and 28.4% (95% CI, 20.5% to 36.3%) of patients from non-*BRCA1/2 *families who were younger than 40 years at first breast cancer developed contralateral breast cancer. Of those who were older than 50 years of age at first breast cancer, 21.6% (95%CI, 12.3% to 30.8%) from families with a *BRCA1 *mutation, 15.5% (95% CI, 7.8% to 23.3%) from families with a *BRCA2 *mutation and 12.9% (95% CI 8.9% to 17.0%) from *BRCA1/2 *negative families developed contralateral breast cancer (Figure 3A, B, C). As expected the highest risks were seen for *BRCA1 *mutation carriers under the age of 35 years with 15.7% (95% CI, 9.7% to 21.7%) after 5 years, 33.4% (95% CI, 24.6% to 42.1%) after 10 years, 45.3% (95% CI, 34.8% to 55.7%) after 15 years and 61.6% (95% CI, 49.0% to 74.1%) after 25 years. Table 2 depicts the cumulative contralateral breast cancer risk estimates for 5, 10, 15, 20, and 25 years after first breast cancer depending on *BRCA1/2 *mutation status and age at first breast cancer.

**Table 2:** Cumulative risks (in %) and 95% confidence intervals (in parentheses) for contralateral breast cancer depending on age at first breast cancer observed in relatives of index patients.

	*BRCA1*	*BRCA2*	*BRCA *negative
Age at first breast cancer < 40 years			
5 years after first breast cancer	14.1 (10.1-18.0)	2.9 (0.0-6.3)	4.8 (2.6-6.9)
10 years after first breast cancer	30.1 (24.0-36.2)	18.2 (7.9-28.5)	10.6 (6.8-14.4)
15 years after first breast cancer	40.8 (33.2-48.3)	20.9 (9.7-32.1)	15.3 (10.4-20.3)
25 years after first breast cancer	55.1 (45.4-64.9)	38.4 (18.5-58.2)	28.4 (20.5-36.3)
Age at first breast cancer 40-49 years			
5 years after first breast cancer	9.2 (5.8-12.5)	6.9 (2.7-11.1)	4.2 (2.9-5.5)
10 years after first breast cancer	16.7 (11.7-21.7)	13.4 (7.0-19.8)	8.4 (6.3-10.5)
15 years after first breast cancer	23.2 (16.9-29.6)	22.0 (12.1-31.9)	10.7 (8.1-13.3)
25 years after first breast cancer	44.5 (33.2-55.7)	40.5 (22.4-58.6)	18.1 (13.9-22.3)
Age at first breast cancer ≥ 50 years			
5 years after first breast cancer	7.1 (3.8-10.5)	3.5 (0.9-6.1)	3.6 (2.7-4.5)
10 years after first breast cancer	11.4 (6.5-16.3)	10.4 (4.9-16.0)	5.5 (4.3-6.7)
15 years after first breast cancer	18.7 (11.0-26.3)	15.5 (7.8-23.3)	8.1 (6.3-9.9)
25 years after first breast cancer	21.6 (12.3-30.8)	15.5 (7.8-23.3)	12.9 (8.9-17.0)
Total			
5 years after first breast cancer	10.4 (8.3-12.5)	4.5 (2.5-6.5)	3.9 (3.2-4.6)
10 years after first breast cancer	20.4 (17.1-23.7)	13.2 (9.2-17.2)	7.1 (6.0-8.2)
15 years after first breast cancer	28.7 (24.4-32.9)	19.0 (13.5-24.4)	9.9 (8.5-11.4)
25 years after first breast cancer	44.1 (37.6-50.6)	33.5 (22.4-44.7)	17.2 (14.5-19.9)

### Other potential modifiers of contralateral breast cancer risk

The number of affected family members, the age at onset of the earliest affected family member, the number of bilateral breast cancers in the family and PBSO were not predictive for the occurrence of contralateral breast cancer. The latter may be due to the fact that only 131 patients of our cohort opted for PBSO. Having only limited information on tamoxifen use which was introduced into breast cancer treatment around 1990, we performed a comparison of women who had their first breast cancer before 1990 with women who had their first breast cancer after 1990. We could not find a significant difference in the time from first to contralateral breast cancer between these two groups.

## Discussion

Little is known about the contralateral breast cancer risk in women with familial breast cancer that tested negative for *BRCA1 *or *BRCA2 *mutations. Despite that lack of knowledge, however, the demand for prophylactic bilateral mastectomy after first breast cancer is increasing not only for mutation carriers but also for women without a *BRCA1 *or *BRCA2 *mutation [14,15]. Therefore, there is an urgent need for empirical data that may allow women to base their informed decision regarding prophylactic bilateral mastectomy on reliable risk estimates.

### Contralateral breast cancer risk depending on mutation status

Our results are concordant with a previous retrospective study on a cohort of 327 familial non-*BRCA1/2 *breast cancer cases [12,13]. These authors found incidences of metachronous contralateral breast cancers after 10 years that were similar to those in sporadic breast cancer patients (that is, 6.4% versus 5.4%, respectively). They also provided evidence that previous reports on higher contralateral breast cancer incidence rates may have been due to a selection bias caused by preferential DNA testing in women with bilateral breast cancer [13]. Moreover, our calculated risks are similar to those reported in the National Cancer Institute's Surveillance, Epidemiology, and End Results (SEER) database, that is, a contralateral breast cancer risk of approximately 2.5% to 12.6% in 10 years depending on estrogen receptor status and age at onset [18,19]. Our data that we estimated for cases from *BRCA1/2 *negative families are in the same risk range of 7.1% (95% CI 6.0% to 8.2%) after 10 years.

Therefore, we conclude that contralateral breast cancer risk for familial non*-BRCA1/2 *breast cancer is essentially in the same range as for women with sporadic breast cancer. However, our risk estimates are in clear contrast with the recently observed increase in prophylactic bilateral mastectomy and prophylactic contralateral mastectomy in women with familial breast cancer and unknown or negative mutation status [14,15,20]. This increase may in part be due to uncertainties and overestimations of the true risks in the case of early age at first breast cancer or due to a positive family history, irrespective of the mutation status [21-23].

The risks that we calculated for relatives of *BRCA1 *or *BRCA2 *mutation carriers are in agreement with our previous report on the same but smaller cohort [5]. Our results are further supported by results from the WECARE study, a population-based, nested case-control study on 705 cases with contralateral breast cancer and 1,398 controls with unilateral breast cancer ascertained through US cancer registries that participated in the SEER registry system [24]. In this study, a woman diagnosed with *BRCA1 *associated breast cancer between the age of 25 to 29 was calculated to have a 5-year and 10-year risk for contralateral breast cancer of 16% and 29%, respectively. This is in line with our results, that is, a risk for contralateral breast cancer of 15.7% (95% CI 9.7% to 21.7%) after 5 years and 33.4% (95% CI 24.6% to 42.1%) after 10 years for women from *BRCA1 *positive families that developed the first breast cancer before the age of 35 years. However, our risk estimates are lower than those recently reported by Metcalfe *et al. *in a cohort of 810 mutation carriers [10] where the authors estimated a 15-year risk of 36.1% for *BRCA1 *and 28.5% for *BRCA2 *mutation carriers. Pierce *et al. *also reported higher risk estimates with a 15-year risk of 39% for *BRCA1 *and *BRCA2 *mutation carriers [3]. The most likely explanation is that these studies focused on index cases or did not exclude index cases, thereby introducing a recruitment bias.

### Modifiers of contralateral breast cancer risk

We present results which highlight that contralateral breast cancer risk in women from *BRCA1/2 *negative families depends on age at onset of first breast cancer, as is the case for women from *BRCA1 *or *BRCA2 *positive families. In a recent paper, Metcalfe *et al. *also confirmed that the risk of contralateral breast cancer depends on age at first diagnosis in *BRCA1/2 *mutation carriers. Women older than 50 years at the time of breast cancer experienced a significantly lower risk of contralateral breast cancer than women diagnosed before the age of 40 years (RR 0.47; 95% CI 0.47 to 0.82, *P *= 0.008) [10]. Metcalfe *et al. *could further show that increasing numbers of affected first degree relatives under the age of 50 years were associated with an increased risk for contralateral breast cancer in *BRCA1 *mutation carriers (RR trend 1.40; 95% CI 1.08 to 1.81, *P *= 0.01) [10]. Despite extensive analyses, we could not confirm this association in our cohort. Moreover, we could not demonstrate an effect of PBSO on contralateral breast cancer risk as described by others [25]. This may be explained by the fact that only 131 women opted for PBSO in this cohort.

### Limitations

While the strength of our study is the large sample size, some limitations should be mentioned. First, we cannot rule out that phenocopies might have been included in our analysis, since only 16% of the relatives from *BRCA1 *or *BRCA2 *positive families were proven mutation carriers. However, Meijers-Heijboer *et al. *calculated a phenocopy rate of 5% to 6% [26]. Therefore, it seems unlikely that phenocopies had a large impact on our results. Second, we cannot exclude a survivorship and recruitment bias. This is supported by other studies which provided convincing evidence for such a bias [13,26].^. ^But as we considered only affected relatives, including deceased patients, it is unlikely for the survivorship and recruitment bias to have a negative impact on our results. Besides, we cannot rule out that the exclusion of index patients from the analysis, which was done to avoid overestimation of risks, may have led to an underestimation to some extent. Third, medical reports could only be obtained from 83% of the patients. This could have led to an incomplete ascertainment of contralateral breast cancer. However, results from a recent population-based study are in line with our risk estimates for contralateral breast cancer in *BRCA1 *and *BRCA2 *carriers [24]. Therefore, it is unlikely that incomplete reporting of affected family members had a considerable influence on our results. Fourth, the stringent selection of high risk families, as illustrated by a 25% mutation prevalence in our cohort, might influence our risk estimates. In countries where selection criteria are different, the risk estimates for the *BRCA1/2 *negative families may vary accordingly. Fifth, we could not demonstrate an effect of further modifying factors such as PBSO, tamoxifen, chemo- and radiation therapy on contralateral breast cancer due to the low uptake of these interventions or incomplete reporting in our cohort [3,7,9-11]. Finally, our analysis did not consider competing cancer events. For instance, the group specific risk estimates may be biased to some extent by the different ovarian cancer risks in the three groups.

## Conclusions

We calculated long term risk estimates for contralateral breast cancer in the largest cohort of women with familial breast cancer reported so far. We demonstrate: 1) that contralateral breast cancer risk for patients from *BRCA1/2 *negative families is low and similar to the risk for patients with sporadic breast cancer; and 2) that contralateral breast cancer risk strongly depends on the mutation status and age at onset of the first breast cancer. This strengthens the importance of genetic testing as a prerequisite for risk estimation and, consequently, informed decision making for or against prophylactic bilateral mastectomy or prophylactic contralateral mastectomy.

## Abbreviations

BRCA: breast cancer gene; HR: hazard ratio; PBSO: prophylactic bilateral salpingo-oophorectomy.

## Competing interests

The authors declare that they have no competing interests.

## Authors' contributions

KR, CE and RS were responsible for the conception and design of the study, acquisition and analysis of data, and drafted the manuscript. SZ, MG, KK, MK, ND, WJ, CM, MG, DV, SP-A, TH, UB, DG, SB and AM acquired data and revised the manuscript critically. All authors read and approved the final manuscript.
